# Correlation of tumor-associated macrophages and clinicopathological factors in Wilms tumor

**DOI:** 10.1186/2045-824X-5-5

**Published:** 2013-03-21

**Authors:** Peter Liou, Leah Bader, Antai Wang, Darrell Yamashiro, Jessica J Kandel

**Affiliations:** 1 Division of Pediatric Surgery, Columbia University Medical Center, 3959 Broadway, CHN 214, New York, NY, 10032, USA; 2 Division of Pediatric Hematology/Oncology/Stem Cell Transplantation, Columbia University Medical Center, New York, NY, 10032, USA; 3 Department of Biostatistics, Columbia University Mailman School of Public Health, New York, NY, 10032, USA

**Keywords:** Wilms tumor, Tumor-associated macrophages, Wilms tumor staging, Lymph node sampling

## Abstract

**Background/purpose:**

Despite high long-term survival rates in patients with Wilms tumor, there is a need to develop better prognostic biomarkers in order to maximize cure while avoiding treatment-associated morbidities. Tumor-associated macrophages have been recently associated with poorer prognosis and increased disease progression in a number of adult cancers. We investigated the relationship between macrophages and clinicopathological fators in this pediatric solid tumor.

**Methods:**

Tissue microarray sections of 124 Wilms tumor cases obtained from the Cooperative Human Tissue Network were stained with CD68, a macrophage marker using standard immunohistochemical techniques and quantified using digital image processing techniques. Macrophage densities were correlated by tumor stage, and survival analyses were conducted with available clinical data. Immunohistochemistry was performed on 25 additional Wilms tumor cases obtained from the tumor bank at Columbia University Medical Center and correlated with presence of tumor microvascular invasion.

**Results:**

Mean macrophage count densities in stage IV tumors were significantly greater than densities in stage I and III tumors (p=.021, .036). Although the overall and disease-free survival did not differ between high and low macrophage presence groups across all stages, increased macrophage presence was associated with decreased disease-free survival in patients with stage II tumors (p=0.035). Increased macrophage presence may have also correlated with decreased disease-free survival in stage IV patients, but the sample size was too small to allow detection of this difference with significance (p=0.575). Increased macrophage presence was associated with tumor microvascular invasion (p=0.0004).

**Conclusion:**

Our results suggest that macrophage recruitment may be associated with disease progression in Wilms tumor. Quantitation of macrophage presence may therefore be a useful adjunct in refining staging algorithms for patients with stage II Wilms tumor. Such data might be useful in the effort to reduce the risk of adverse effects associated with under- or overtreatment of this neoplasm.

## Introduction

Wilms Tumor is the most common pediatric renal malignancy, and the second most common abdominal solid tumor in children [1]. There are approximately 500 new cases diagnosed in the United States each year. Due to the development of evidence-based treatment protocols and their refinement by the multidisciplinary cooperative groups over the last 40 years, long-term survival rates in Wilms tumor have dramatically improved [2]. With these advances, a focus on refining therapy to reduce adverse effects while maintaining excellent outcomes has become increasingly important. However, an array of biomarkers that may be useful in this refinement are still lacking. Thus, new biomarker discovery could reduce both overtreatment of low-risk tumors and undertreatment of high-risk tumors, limiting unnecessary morbidity and undesired outcomes in these children.

Treatment of Wilms tumor in North America is currently based upon tumor staging, the presence of favorable or unfavorable histology, and age, using protocols developed by the Children’s Oncology Group (COG) [3,4]. Traditionally, tumor stage has correlated closely with disease-free and overall survival [5]. Accurate tumor staging is therefore critical to determining appropriate treatment. Stage I and II tumors are amenable to surgical resection with or without limited chemotherapy, while Stage III and IV tumors require more intensive treatment, including extended adjuvant chemotherapy regimens and in some cases, radiation therapy.

The diagnosis of stage II Wilms tumor offers a particular prognostic challenge, as these children may be at higher risk of understaging. Some authors have suggested that this depends on the number of lymph nodes sampled. In a large retrospective study, Kieran *et al.* found that the likelihood of finding a positive lymph node, and thus upstaging to stage III, was greater when more than 7 were sampled, suggesting that insufficient sampling may limit the accuracy of stage determination [6]. Understaging could therefore lead to inadequate treatment of Stage III tumors, with consequent poorer outcomes [7,8]. Stage II patients in whom lymph nodes were not sampled experienced an increase in local tumor recurrence, again associated with poorer outcomes [9]. Consequently, there is a clear need to identify better prognostic predictors in patients with Stage II tumors.

Recently, tumor-associated macrophages (TAMs) have become the focus of significant interest for their ability to predict tumor prognosis in a number of malignancies [10]. As cellular effectors of the innate immune system, macrophages play essential roles in a myriad of processes, including immune response, inflammation, tissue remodeling, and injury repair [11]. Macrophages are also major constituents of tumor stroma, and an emerging body of evidence suggests that they play a prominent role in tumor growth and survival. In particular, M2 (alternatively activated) macrophages secrete anti-inflammatory cytokines, promote tissue repair/remodeling, angiogenesis, and elicit downregulation of T-cells and other immune effectors [12,13]. Similarly, a number of experimental studies have demonstrated the ability of neoplastic cells to recruit M2 macrophages, which supports tumor growth, stimulates tumor angiogenesis, suppresses host immunity, and promotes invasion and metastasis [14,15]. Thus, TAMs have attracted significant interest both as biomarkers and as potential targets for novel therapies [13].

A number of clinical studies have previously linked macrophage presence and prognosis in a variety of adult human cancers, including breast, endometrial, renal cell carcinoma, poorly differentiated thyroid cancer, and Hodgkin’s lymphoma [16-20]. In most prior reports, high numbers of macrophages have been correlated with worse prognosis, more rapid tumor progression, and decreased disease-specific survival [10]. Consistent with these observations, tumor progression and metastasis are significantly reduced in macrophage-deficient experimental models [21]. Taken together, these data suggest that macrophage presence might also be useful as a biomarker in pediatric solid tumors. In this study, we sought to examine the relationship between macrophage presence and clinicopathological factors in Wilms tumor.

## Methods

### Immunohistochemistry

Tissue microarrays (TMAs) containing 154 unique Wilms tumor cases were obtained from the Cooperative Human Tissue Network tissue bank (CHTN, Columbus, OH), by application to the Renal Tumors Biology Subcommittee of the Children’s Oncology Group (COG). Each case includes two to four 1-millimeter cores of paraffin embedded tissue mounted onto the TMA in random order. 27 cases were removed from analysis due to incomplete clinical information. An additional 3 cases containing stage V disease were eliminated, leaving 124 for final analysis.

TMAs were stained for CD68 using standard immunohistochemistry protocols. Slides were warmed at 40°C overnight and deparaffinized in xylene, followed by rehydration in decreasing concentrations of ethanol. Antigen retrieval was carried out with target retrieval solution pH9 (DAKO, Carpinteria, CA) for 15min in a steamer at 100°C, then allowed to cool to room temperature for 20min. The slides were blocked for 30min at room temperature with CAS blocking reagent (Invitrogen, Carlsbad, CA) to reduce non-specific staining. Mouse anti-human CD68 monoclonal antibodies (clone PG-M1, DAKO), diluted 1:200 in CAS block were incubated with the slides overnight at 4°C. After thorough rinsing with phosphate-buffered saline, biotinylated anti-mouse secondary antibodies were incubated with slides at room temperature for 30min. Development of the stain was performed using 3,3’-Diaminobenzidine (DAB) chromogen (Vector Laboratories, Burlingame, CA) according to manufacturer specifications. Tonsillar tissue was used as positive control.

### Macrophage counting

**Figure 1:**
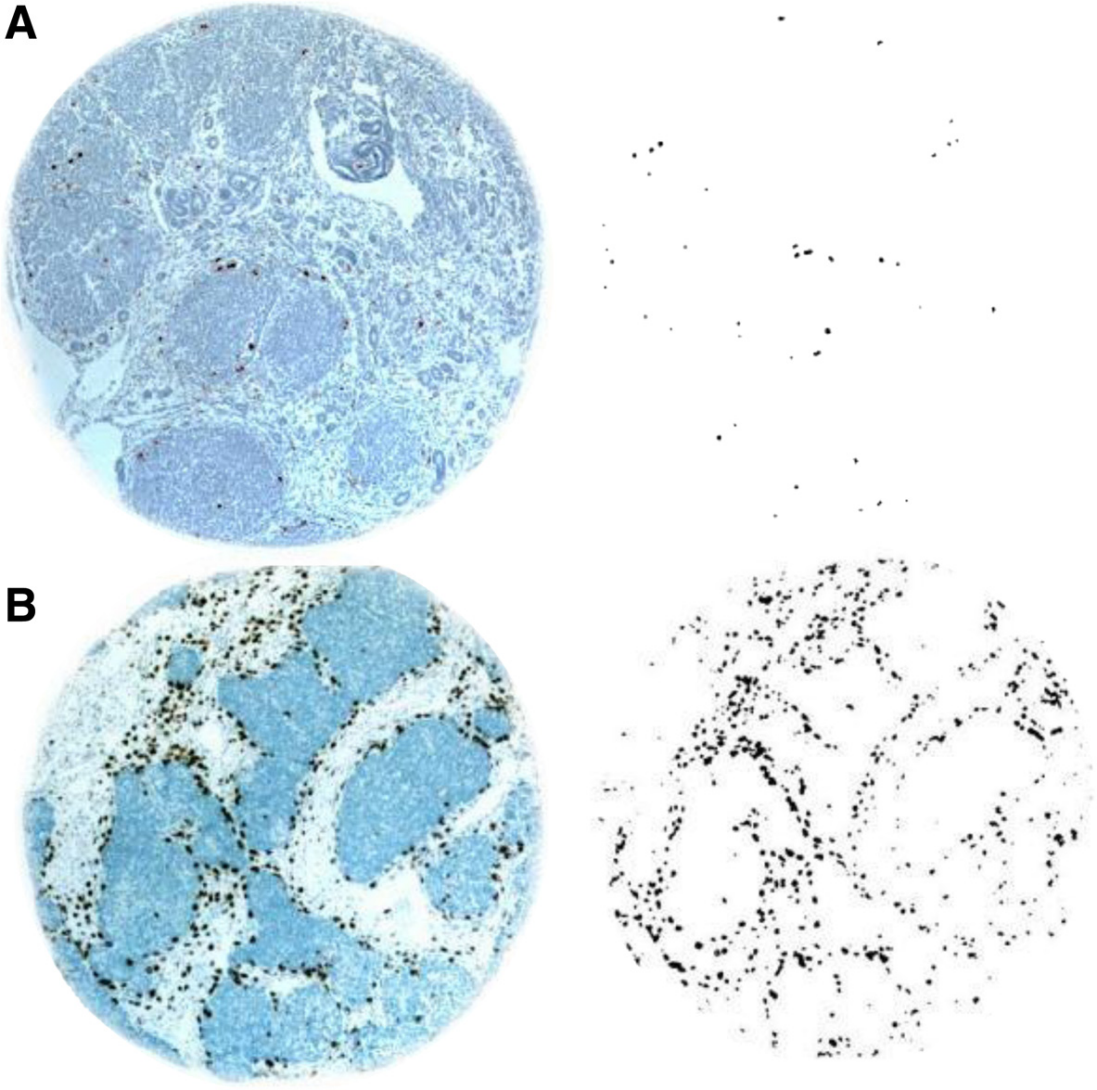
**High and Low Macrophage Count Density Wilms Tumor Tissue Cores. (A)** Representative example of a low macrophage count density Wilms’ Tumor tissue core, before and after image processing. **(B)** Representative example of a high macrophage count tissue core.

High-resolution digital images were taken of each tissue core at 4× magnification using a digital microscope camera (Diagnostic Instruments, Sterling Heights, MI) mounted on a Nikon E600 microscope. Images were then loaded into Photoshop CS3 (Adobe Systems, San Jose, CA) with scientific image processing software (Reindeer Graphics, Asheville, NC). Although the size of tissue cores was estimated to be 1mm in diameter, we accurately measured the size of each core in pixels using the *magnetic lasso* feature to accommodate minor variation. The image threshold was then set to eliminate the core background and isolate the immunostained macrophages. To separate closely adjacent macrophages and eliminate non-specific background noise, the *Binary Image Morphology* function was set to *Open*, with a coefficient of 4 and depth of 2. Background noise was further reduced by eliminating individual spots containing less than 6 pixels (Figure 1). Discrete macrophages were counted using the *Count* feature. A count density per individual case was then calculated using the following formula:

$$\mathrm{Count}\;\mathrm{Density}=\frac{\mathrm{Average}\;\mathrm{Macrophage}\;\mathrm{Number}}{\mathrm{Average}\;\mathrm{Core}\;\mathrm{Area}\;\left(\mathrm{Pixels}\right)}*\mathrm{Constant}$$

The average macrophage number was calculated as the mean number of macrophages in all tissue cores per individual case. An arbitrary constant was used to facilitate ease of analysis and scoring.

### Statistical analysis

ANOVA and respective post-hoc T-tests using the Tukey criteria were performed on the count densities of all Wilms tumor cases, stratified by overall tumor stage. To determine associations between count density and disease free and overall survival, analyses were performed using the Logrank test. We chose a count density less than 10 to represent a low macrophage presence, and a count density greater than 10 to represent high macrophage presence. Separate categorical analysis using Fisher’s exact test was used to corroborate significant associations found between count density and survival.

### Macrophage presence and microvascular invasion

Due to the lack of pathologic data for tissues obtained from the CHTN, we were unable to assess the presence of microvascular invasion in the TMA cases. Therefore, we acquired 25 separate Wilms tumor cases from the Columbia University Medical Center tumor bank, with accompanying de-identified pathology reports. Because the samples contained much more tissue than the cores available on a tissue microarray, the macrophage counting method was adjusted. Images were taken of ten macrophage hot-spots (areas within the tissue containing the highest density of macrophages) per Wilms tumor case, at 20x magnification. The images were then loaded into Photoshop CS3 and macrophages were counted using the same methods described above. The average counts of three images containing the greatest number of macrophages per Wilms tumor case were recorded and correlated against presence of microvascular invasion. Count densities were not required, as the images taken were the same size. Statistical analysis was performed using Fisher’s exact test.

## Results

### Patient characteristics

A total of 154 unique Wilms tumor cases with accompanying tissue cores were provided by the Children’s Oncology Group. 124 cases were selected for final analysis, as 30 were either missing corresponding clinical information or were stage V tumors. 47 patients had overall stage I tumors, 20 were stage II, 45 were stage III, and 12 were stage IV. Two patients had received chemotherapy prior to surgery: one was designated as stage III, the other as stage IV. The mean age at time of surgery was 39.7 months (Range 2–120, Stdev 29.5). 53% of patients were female and 47% were male. 60% patients were Caucasian, 25% were African American, 13% were Hispanic, and 2% Other. The overall cumulative long-term survival rate was 87.4% and disease free survival rate was 71.1% (See Additional file 1). Stage IV tumors were associated with both decreased overall and disease-free survival. Additionally, stage II tumors were associated with decreased disease-free survival.

### Macrophage counts and Wilms tumor staging

We chose to immunostain for the CD68 macrophage marker, the most widely used antigen in both clinical and research settings. Additionally, its low cost and relative ease of staining compared to other markers makes CD68 attractive as a candidate prognostic biomarker for patient use. A relative limitation to the use of this marker may be the need to confirm specificity [22]. However, in our study, cells that positively stained for CD68 were subsequently confirmed to exhibit classic histological characteristics of macrophages by two independent pathologists. Tissue microarrays obtained from the CHTN were used to allow for rapid processing and analysis of 124 Wilms tumor cases.

**Figure 2:**
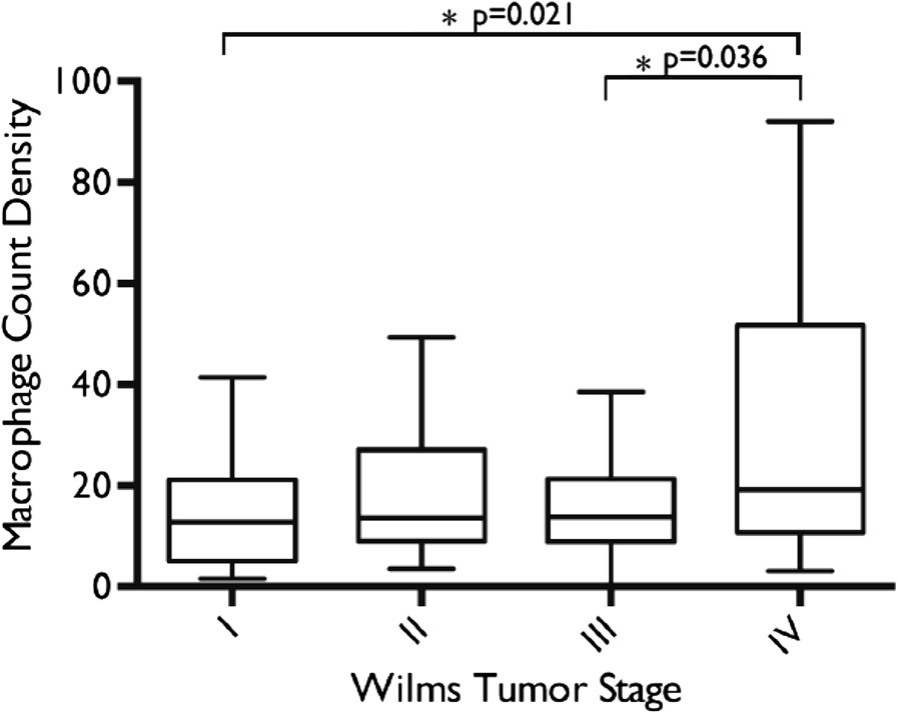
**Comparison of Mean Macrophage Count Densities by Tumor Staging.** ANOVA demonstrated a significant difference in mean macrophage count densities when stratified by tumor stage. Post-hoc T-test analysis using Tukey’s method showed a significant difference in mean macrophage count density between stage IV and stage I, and stage IV and stage III tumors.

ANOVA demonstrated a significant difference in macrophage count densities when stratified by tumor staging (p=0.029). Post hoc T-test analysis using Tukey’s method showed a significant difference in mean macrophage count density between stage IV and I, and stage IV and III tumors (Figure 2). There was no significant difference in mean count densities between stage IV and II tumors. Mean count densities in stage II, III, and IV tumors were significantly greater than the mean count density measured in normal kidney tissue as control.

### Macrophage presence and disease free/overall survival

**Figure 3:**
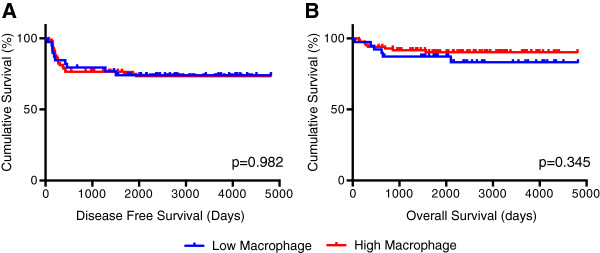
**(A) Disease-free and (B) overall survival comparisons of patients with low and high macrophage count densities.** Logrank analysis was used to compare survival differences between the two populations.

**Figure 4:**
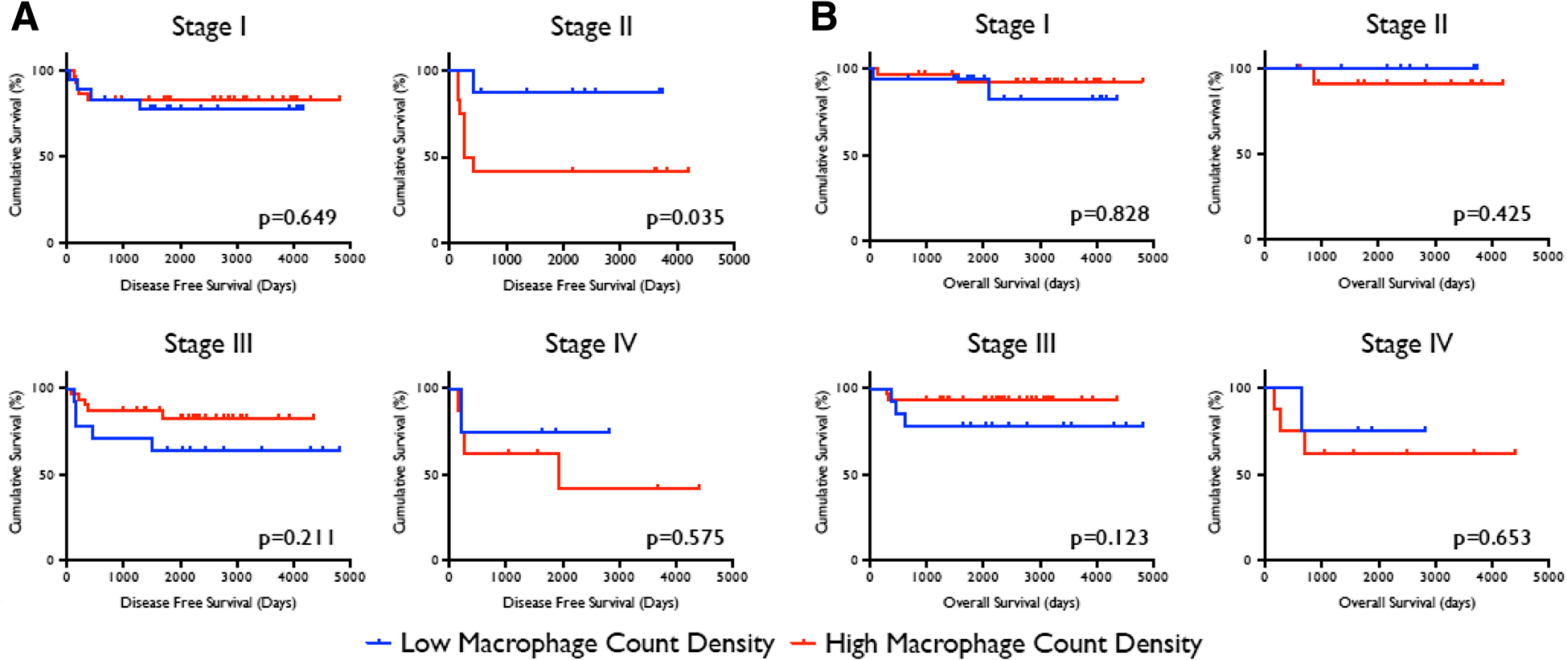
**(A) Disease-free survival and (B) overall survival comparisons of patients with high and low macrophage count densities, stratified by Wilms Tumor staging.** Logrank analysis was used to compare survival between the two populations.

Cases with a mean macrophage count density 10 or less were considered as having a low macrophage presence, and those with mean count densities greater than 10 having a high macrophage presence. We selected this cutoff for macrophage presence as a reasonable number for general pathologic use. Overall survival and disease-free survival did not significantly differ between low and high macrophage presence groups (Figure 3). However, in patients with stage II tumors, greater macrophage presence was associated with decreased disease-free survival (p=0.035, Figure 4). This observation was independently corroborated with a Fisher’s Exact Test demonstrating a significant association between greater macrophage presence and disease relapse (p=0.01). Patients with stage IV tumors that have higher macrophage presence also appear to have decreased disease free survival (Figure 4), but the study was inadequately powered to detect a significant difference (p=0.575). Overall survival did not differ significantly between low and high macrophage presence groups when tumor stages were analyzed independently.

### Macrophage count and microvascular invasion

25 Wilms Tumor cases obtained from the Columbia University Medical Center Tumor Bank were stained for macrophages and correlated with presence or absence of microvascular invasion. Samples were considered having high macrophage counts if the mean number of macrophages per high-powered field was greater than 50. Increased macrophage counts were significantly associated with microvascular invasion (p=0.0004).

## Discussion

In this report, we provide the first clinicopathologic examination of macrophage presence in a pediatric solid tumor. We used tissue microarrays to conduct our analyses, as they have been previously validated as a method of cohorting larger sample numbers and allowing for more robust analysis of immunohistochemical signal [16]. In prior studies, individual macrophages have been manually counted or scored by blinded investigators [16,18,20,23], which can lead to inconsistent results. To avoid this, we used an objective macrophage quantitation method that provides for consistency and ease of analysis. Our method employs digital image capturing and processing to produce a macrophage “count density” that can be easily interpreted and potentially utilized in the clinical setting.

In our cohort of 124 Wilms tumor cases, we demonstrated that the mean macrophage count density in stage IV tumors was significantly higher than that of stage I and III tumors. This finding indicates that increased macrophage presence correlates with tumor stage, the primary clinical factor directing choice of therapy. Intriguingly, sub-group analysis by tumor stage demonstrated the macrophage density was significantly different when disease-free survival in stage II patients was studied, although overall survival did not differ between high and low macrophage presence groups. Using an independent set of samples from 25 patients, we also showed that increased macrophage count is associated with the presence of tumor microvascular invasion, supporting future investigation into the mechanism by which macrophage recruitment promotes progression in Wilms tumor.

Tumor-associated macrophages have been shown experimentally to promote disease progression by supporting inflammation and tumor cell survival [24]. Recent studies also suggest that TAMs play a major role in activating the “angiogenic switch,” stimulating tumor angiogenesis [10]. Our study is consistent with this concept, and suggests an association between increased macrophage infiltration and the presence of microvascular invasion by tumor cells. The mechanisms of disease promotion by TAMs in pediatric solid tumors have not been previously explored. Our study was limited by the small amount of pre-selected tissue cores per case used in immunohistochemistry, which could have underrepresented the true number of macrophages in the tumor, as well as the limited number of cases that is typical for children’s cancers. However, our findings achieved statistical significance despite these constraints, suggesting that this may be a useful approach in the setting of children’s cancers. Finally, although our study will need prospective validation, we show that a relatively simple technique might be an additional tool for prognosticating Wilms tumor cases during pathological review. Our study was conducted with the approval and support of the Renal Tumors Biology subcommittee of the Children’s Oncology Group, the body which can implement systematic biomarker study for pediatric solid tumors.

In summary, our findings suggest that macrophage presence may be a useful adjunct in refining staging algorithms for patients with pathologic stage II Wilms tumor. As noted previously, these patients may be at risk for sampling error and undertreatment. Examination for macrophage presence in tumor samples could be used to complement current pathologic assessments to guide staging. By refining prognostic stratification between stage II and III tumors, the risk of adverse effects associated with under- or overtreatment may be reduced. A similar trend in stage IV patients did not achieve statistical significance due to the small number of cases, and may also be worthy of further examination. Increased macrophage presence was significantly associated with tumor microvascular invasion, consistent with prior reports of TAM function in other tumor systems. Taken together, these data support further study of the role of macrophages in the pathobiology of pediatric Wilms tumor.

## Competing interests

The authors declare that they have no competing interests.

## Authors’ contributions

PL participated in study design, immunostaining, data analysis, and drafted the manuscript. LB participated in immunostaining. AW assisted in statistical analysis. DY assisted in image processing and manuscript editing. JJK conceived of the study and participated in study design and manuscript editing. All authors read and approved of the final manuscript.

## Supplementary Material

Additional file 1 Supplemental Data. Click here for file

## Support

PL and LB were supported by grants from the Doris Duke Foundation for Clinical Research. AW is supported by a Columbia University Irving Cancer Research Center support grant. DY received support from NIH 1R01CA124644. JJK received support from NIH 1R21EB015040, the Pediatric Cancer Foundation, and the Sorkin Fund.
