# Supplementary figures and images for: Correlation of tumor-associated macrophages and clinicopathological factors in Wilms tumor
Source: Vasc Cell. 2013 Mar 21;5:5. doi: 10.1186/2045-824X-5-5 (PMC3610208; doi:10.1186/2045-824X-5-5)

## Slide 1
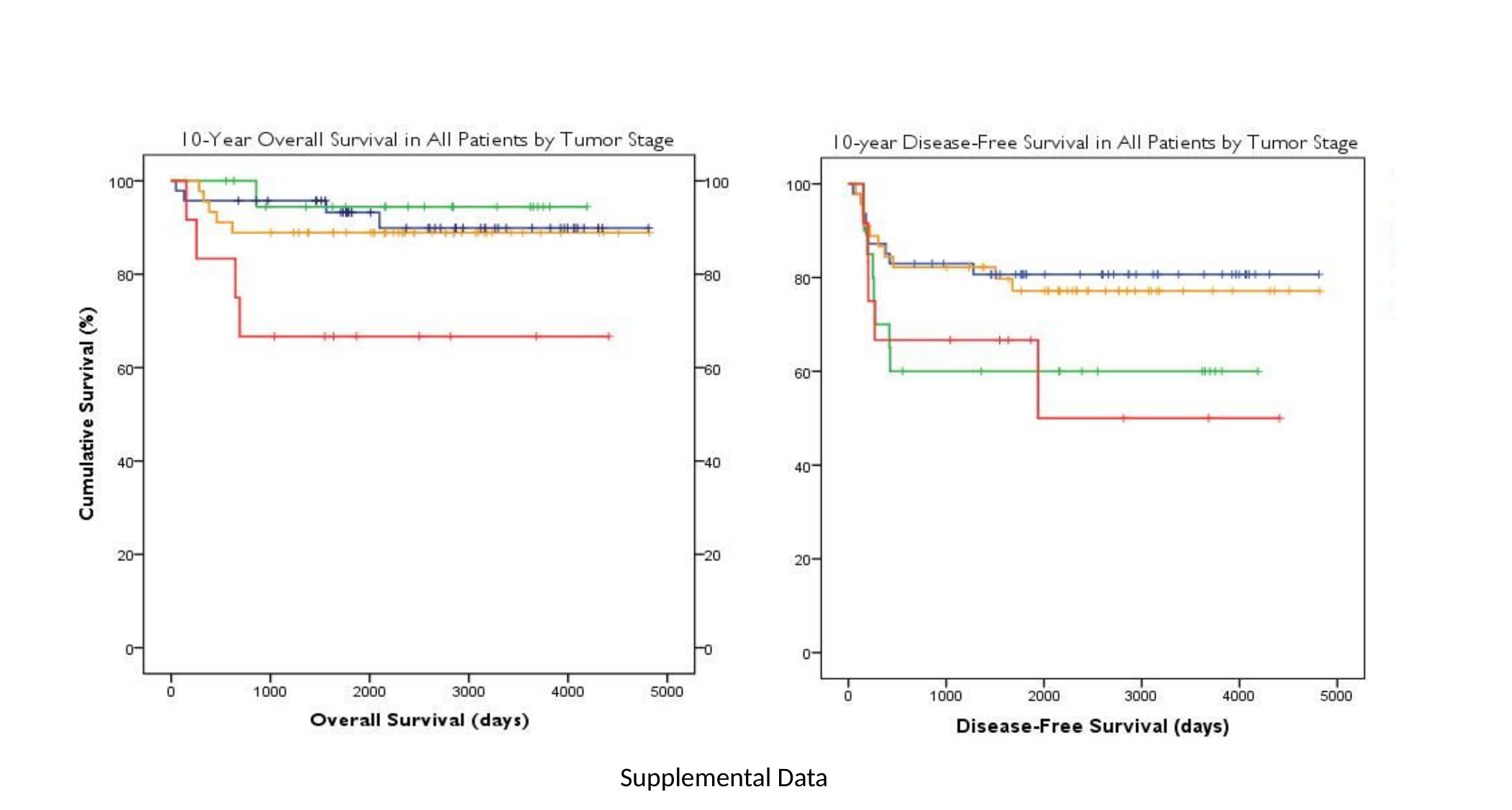

Supplemental Data

Supplement: Additional file 1 — Supplemental Data. [file 2045-824X-5-5-S1.pptx]
